# The Modifying Effect of Obesity on the Association of Matrix Metalloproteinase Gene Polymorphisms with Breast Cancer Risk

**DOI:** 10.3390/biomedicines10102617

**Published:** 2022-10-18

**Authors:** Nadezhda Pavlova, Sergey Demin, Mikhail Churnosov, Evgeny Reshetnikov, Inna Aristova, Maria Churnosova, Irina Ponomarenko

**Affiliations:** Department of Medical Biological Disciplines, Belgorod State National Research University, 308015 Belgorod, Russia

**Keywords:** *MMP*, breast cancer, obesity, SNP, association

## Abstract

Objective: We investigated the possible modifying effect of obesity on the association of matrix metalloproteinase (*MMP*) gene polymorphisms with breast cancer (BC) risk. Methods: A total of 1104 women divided into two groups according to their body mass index (BMI): BMI ≥ 30 (119 BC, and 190 control) and BMI < 30 (239 BC, and 556 control) were genotyped for specially selected (according to their association with BC in the previous study) 10 single-nucleotide polymorphisms (SNP) of *MMP1*, *2*, *3*, *8*, and *9* genes. Logistic regression association analysis was performed in each studied group of women (with/without obesity). Functional annotation of BC-correlated *MMP* polymorphic variants was analyzed by in silico bioinformatics. Results: We observed significant differences in the involvement of *MMP* SNPs in BC in obese and non-obese women. Polymorphic loci *MMP9* (c.836 A > G (rs17576) and c. 1721 C > G (rs2250889)) were BC-protective factors in obese women (OR 0.71, allelic model, and OR 0.55, additive model, respectively). Genotypes TT *MMP2* (c.-1306 C > T,rs243865) and AA *MMP9* (c. 1331-163 G > A,rs3787268) determined BC susceptibility in non-obese women (OR 0.31, and OR 2.36, respectively). We found in silico substantial multidirectional influences on gene expression in adipose tissue BC-related polymorphic loci: BC risk allele A-rs3787268 in non-obese women is associated with low expression *NEURL2*, *PLTP*, *RP3-337O18.9*, *SPATA25*, and *ZSWIM1*, whereas BC risk allele A-rs17576 in obese women is associated with high expression in the same genes in visceral and/or subcutaneous adipose. Conclusions: our study indicated that obesity has a significant modifying effect on the association of *MMP* genes with BC risk in postmenopausal women.

## 1. Introduction

Breast cancer is a malignant tumor that is formed from the epithelial structures of the breast [1]. According to the data published in 2020 by the International Agency for Research on Cancer, BC is the most frequently diagnosed cancer in women in comparison with all other types of oncopathology; it accounts for 24.5%, and 2.3 million new cases of the disease are registered annually in the world [2]. In the structure of mortality of the female population in the world as a whole, the BC proportion is 15.5% (annually this malignant tumor is the cause of death of 685,000 women) [3].

According to modern ideas, hereditary factors play an important role in BC formation [4,5,6]. Based on large-scale twin studies conducted in European populations, it was shown that the cause of almost one third of all cases of the disease (31%) is genetic factors [4]. As a result of the BC genome-wide association study (GWAS), about 200 different genetic factors (polymorphisms) have been identified with the disease (GWAS catalog data), which are associated with 18% of BC heritability [5]. Thus, a significant number of the hereditary factors (≈40%) determining BC development remain unknown to date, which determines the relevance of further research on this theme.

Among the BC candidate genes whose connection with the disease *a priori* have a “serious” pathophysiological justification, the genes of *MMP* occupy a special place [7,8,9]. Proteins controlled by these genes, due to their pronounced functional effects aimed at the processes of remodeling of the extracellular matrix [10], are important in BC biology, affecting the tumor growth and metastasis processes [10,11]. Convincing data have been obtained on the association of the production of a number of MMPs in the lesion with the induction of tumor growth and invasion, its more active metastasis, and poor survival in patients with BC [7,10,12]. However, at the same time, despite a fairly large number of association studies on the relationship of *MMP* gene polymorphism conducted at the moment (the PubMed/MEDLINE database contains materials from more than 50 such studies), the results of these studies are ambiguous and often do not agree with each other [13,14,15,16,17,18,19], etc. For example, among four genetic studies of the association of c.259 T > C (rs1940475) *MMP8* with BC, only one study established its association with the disease (BC metastasis) [13], whereas in three studies reliable data on the involvement of this polymorphism in BC formation were not obtained [14,15,16].

The literature data indicate a relation between BMI and BC [20,21,22,23,24]. However, in spite of the considerable accumulated factual material on this problem, there is no unambiguous assessment of the nature of the link between BMI and BC in the modern literature. A number of studies have shown positive correlations between BMI and BC [20,21,23]. For example, a meta-analysis conducted by Liu et al. showed an increase in BC risk of 2% for each 5 kg/m^2^ BMI increased [23]. On the contrary, in several other studies, including those based on Mendelian randomization, negative associations between BMI and BC have been demonstrated [22,24]. It is hypothesized that the premenopausal/postmenopausal status of patients and the BMI-associated estrogen level in these women has an immediate influence on the association features of BMI with BC [23,24]. Therefore, it is obvious that BMI has a significant impact on susceptibility to BC, but the nature/mechanism (including genetic) of this relationship is not fully understood.

At the same time, it is important to emphasize that despite: (a) BMI involvement in BC formation [20,21,22,23,24], (b) a significant association of the *MMP* gene polymorphic loci with BC [13,17,18,19], and (c) correlations of *MMP* gene polymorphisms and the MMP protein level with obesity and obesity-related phenotypes (blood pressure, endothelial dysfunction, metabolic syndrome, etc.) [25,26,27,28,29], an integrated comprehensive genetic and bioinformatic analysis of the *MMP* gene association features with BC in obese/non-obese women has not been carried out.

We investigated the possible modifying effect of obesity on the association of *MMP* gene polymorphisms with BC risk.

## 2. Materials and Methods

### 2.1. Study Subjects

This «case-control» study was approved by the Local Medical Ethical Committee of the Belgorod State University of Russia, and signed informed consent was obtained from all participants. A total of 1104 women were divided into 2 groups according to their BMI: BMI ≥ 30 (119 BC, and 190 control) and BMI < 30 (239 BC, and 556 control) were consecutively recruited between March 2010 and December 2016 at the Belgorod Regional Oncological Dispensary (BC group) and Saint Ioasaph Belgorod Regional Clinical Hospital (control group) of Russia. Body Mass Index was computed as proportion of weight of body (in kilograms) to height (in squared meters) (kg/m^2^). We used well-known World Health Organization categories of BMI: < 18.5 (underweight), 18.5–24.9 (normal weight), 25.0–29.9 (overweight), and ≥30 (obese). Breast cancer was diagnosed by histological examination. The control group had no BC, cancer history, or clinically serious vital organ disorder. All cases and control group women were born (living) in the Central Region of the Russian Federation and were Caucasian [30,31].

### 2.2. Single-Nucleotide Polymorphism Selection and Genotyping

Genomic deoxyribonucleic acid (DNA) was extracted from peripheral venous blood samples (≈4–5 mL) [32] according to previously presented phenol-chloroform protocol [33]. Purity and concentration of DNA samples were assessed by spectrophotometer (Nanodrop 2000, Thermo Scientific, Waltham, MA, USA) [34].

Ten polymorphic loci of the five *MMP* genes, such as c.-1607 2G > 1G (rs1799750) *MMP1*, c.-1306 C > T (rs243865) *MMP2*, c.133 C > T (rs679620) *MMP3*, c.259 T > C (rs1940475) *MMP8*, c.-1562 C > T (rs3918242) *MMP9*, c.139-369 T > C (rs3918249) *MMP9*, c.836 A > G (rs17576) *MMP9*, c. 1331-163 G > A (rs3787268) *MMP9*, c. 1721 C > G (rs2250889) *MMP9*, and c.2003 G > A (rs17577) *MMP9* were selected from previous BC association studies (Supplementary Table S1) [13,14,15,16], etc., and the assessment of their functional value (Supplementary Table S2) [26,27,29,35,36,37,38].

The CFX96 Real-Time polymerase chain reaction System (company of Bio-Rad) was used for DNA sample genotyping; the experimental data obtained were managed using CFX-Manager™ Software [39]. Duplicate testing of a randomly selected five percent of DNA samples was performed for quality control of the experimental data [40]. The data of re-genotyping almost completely coincided with the data of primary genotyping (the error rate was 0.5%).

### 2.3. Statistical and Bioinformatics Analysis

We conducted a comparative analysis between the observed and expected (according to the Hardy–Weinberg equilibrium (HWE)) allele/genotype frequencies for all considered loci among patients and in the control in two studied groups (BMI ≥ 30, and BMI < 30). For the purpose of evaluation of the possible modifying effect of obesity on the association of *MMP* gene polymorphisms with BC risk, we performed association analysis separately in each studied group of women (BMI ≥ 30, and BMI < 30). Covariates included in the logistic regression association models (additive, recessive, dominant, allelic) were age, and BMI. All statistical genetic computations were executed using gPLINK (Java-based software package) [41], resulting in calculated parameters of odd ratios and their confidence intervals (OR and 95% CI, respectively) [42]. In our work, an adaptive permutation testing (for correction, for multiple comparisons) was made [43]. Taking into account the results of the permutation test, the *p_perm_* level of <0.05 was considered statistically significant [44].

The functional annotation of BC-correlated *MMP* polymorphic variants was analyzed by in silico bioinformatics [45,46]. Based on publicly available databases such as HaploReg, GTExportal, PolyPhen-2, SIFT, and GeneMANIA [47,48,49,50] widely used in genetic research, we appreciated eQTL (expression quantitative locus), sQTL (splicing quantitative locus), and epigenetic, non-synonymous effects of BC-associated SNPs [51,52], including the functional effects of these loci in adipose tissue.

## 3. Results

The baseline (phenotypic) parameters of case (BC) and control (cancer-free) subjects in both examined BMI-difference groups (BMI ≥ 30, and BMI < 30) are listed in Table 1. The average age of both BC patients and control individuals with BMI ≥ 30 was much higher (by more than five years) compared to individuals with BMI < 30, due to a higher proportion of subjects aged ≥50 years old among them (by 1.16 times) (Table 1). Also, among individuals with a BMI ≥ 30, the percentage of postmenopausal women was ≈10% higher and reached 74 (control)—75 (case) %. The patients’ BMI was higher than in the control in both investigated cohorts (BMI ≥ 30, and BMI < 30). Importantly, patients with BC, compared with the control, both in the BMI ≥ 30, and BMI < 30 groups, had higher fasting blood glucose, total cholesterol (TC), low-density lipoprotein cholesterol (LDL-C), and triglyceride (TG) levels, and lower high-density lipoprotein cholesterol (HDL-C) (Table 1). At the same time, in both the BC and control groups with BMI ≥ 30, the parameters of blood glucose, TC, LDL-C, and TG were increased and parameter HDL-C was decreased in comparison with similar indicators in patients and controls with BMI < 30. Both BC-studied groups (BMI ≥ 30 and BMI < 30) were dominantly subjected to the T_0_-T_2_ clinical tumor stage (79% and 72%, respectively), and the main pathological cancer parameters were ductal carcinoma (95%, and 94%), estrogen receptor (ER) positive (69%, and 64%), progesterone receptor (PR) positive (62%, and 57%), and histological grade G1/G2 (70%, and 67%) (Table 1).

The materials presented in Supplementary Table S3 (BMI < 30 subjects) and Supplementary Table S4 (BMI ≥ 30 subjects) show minor allele frequencies and genotype distribution of investigated SNPs in both BC and BC-free groups, and demonstrate they satisfy the Hardy–Weinberg equilibrium (Bonferroni correction for the number of studied loci is introduced; the threshold value of *p_bonf_* is at least 0.005(0.05/10)).

We found differences in the link of the examined *MMP* genes’ polymorphic loci with BC in the studied groups with BMI < 30 and BMI ≥ 30. Two SNPs of the *MMP2* and *MMP9* genes (c.-1306 C > T,rs243865, and c. 1331-163 G > A,rs3787268, respectively), were associated with the disease among BMI < 30 subjects, and the other two loci of the *MMP9* gene (c.836 A > G,rs17576, and c. 1721 C > G,rs2250889) were disorder-correlated among the BMI ≥ 30 women (Table 2).

The individuals carrying the minor c.-1306 TT (rs243865) *MMP2* genotype had a 69% lower BC risk than individuals with the c.-1306 CC + CT (rs243865) *MMP2* genotypes (recessive model, OR*_recessive_* = 0.31 95% CI*_recessive_* 0.09–0.99 *p_recessive_* = 0.043 *p*_perm(*recessive*)_ = 0.045), while the women carriers of the minor c.1331-163 AA (rs3787268) *MMP9* genotype had a more than two times higher risk of BC than subjects with 1331-163 GG + GA (rs3787268) *MMP9* genotypes (recessive model, OR*_recessive_* = 2.36 95% CI*_recessive_* 1.12–4.97 *p_recessive_* = 0.024 *p*_perm(*recessive*)_ = 0.025) among BMI < 30 individuals (Table 2).

Among BMI ≥ 30 subjects, BC protective alleles were c.836 G (rs17576) and c. 1721 G (rs2250889) *MMP9*. The individuals carrying these minor allelic variants had a 29% (allelic model, OR*_allelic_* = 0.71 95% CI*_allelic_* 0.50–1.00 *p_allelic_* = 0.047 *p*_perm(*allelic*)_ = 0.049) and 45% (additive model, OR*_additive_* = 0.55 95% CI*_additive_* 0.31–0.98 *p_additive_* = 0.042 *p*_perm(*additive*)_ = 0.043) lower BC disorder risk, respectively, than individuals with reference alleles in these loci (A,rs17576 and C,rs2250889) (Table 2). Haplotype analysis with covariate adjustment and permutation procedure revealed no significant associations in either the BMI ≥ 30 or the BMI < 30 cohorts (*p_permut_* > 0.05).

### In Silico Functionality Analysis, BC Involved SNPs

*Breast cancer-associated SNPs in the BMI < 30 group.* According to HaploReg bioinformatic data, both BC-correlated *MMP* SNPs, c.-1306 C > T (rs243865) *MMP2*, and c. 1331-163 G > A (rs3787268) *MMP9*, possessed weighty epigenetic effects (Supplementary Table S2) and had the potential to influence chromatin structure (DNase footprint/peaks), transcription factors Myf (rs243865) and Sox,HDAC2,Mef2,Pou1f1,p300,Zfp105 (rs3787268) binding (Supplementary Table S5) and transcriptional regulation by promoter/enhancer. Importantly, c.-1306 C > T (rs243865) *MMP2* was placed in DNA promoter/enhancer regions in adipose culture cells: adipose nuclei (Epigenome ID: E063, Mnemonic: FAT.ADIP.NUC), adipose-derived mesenchymal stem cell cultured cells (Epigenome ID: E025, Mnemonic: FAT.ADIP.DR.MSC), and mesenchymal stem cell-derived adipocyte cultured cells (Epigenome ID: E023, Mnemonic: FAT.MSC.DR.ADIP) (HaploReg data). Also, c. 1331-163 G > A (rs3787268) *MMP9* had a likely impact of transcriptional regulation in adipose nuclei (this SNP is localized in the DNA enhancer site).

Data extracted from GTE x portal demonstrated that both BC-related SNPs, c.-1306 C > T (rs243865) *MMP2*, and c. 1331-163 G > A (rs3787268) *MMP9*, are expression quantitative trait loci, and defined mRNA production levels of nine genes: *ZSWIM1*, *ZNF335*, *SLC12A5*, *RP3-337O18.9*, *PLTP*, *NEURL2*, *CD40*, *SPATA25* (rs3787268), and *RP11-212I21.2* (rs243865) (Supplementary Table S6). It is important that seven of these nine genes are expressed in adipose tissue, including adipose–visceral (omentum) (*ZSWIM1*, *SLC12A5*, *RP3-337O18.9*, *NEURL2*, *SPATA25*) and adipose–subcutaneous (*SLC12A5*, *RP3-337O18.9*, *PLTP*, *NEURL2*, *CD40*). Interestingly, BC-risk minor allele c.1331-163 A (rs3787268) *MMP9* is associated with low expression in adipose tissue of the absolute majority of the above genes (β = −0.14–−0.40), and only the eQTL effect of this allele on the *CD40* expression is positive (β = 0.18) (Supplementary Table S6). Besides this, allelic variant c.1331-163 A (rs3787268) *MMP9* has a direct correlation with *PLTP* splicing level in adipose–subcutaneous (β = 0.36) and breast–mammary tissue (β = 0.41) (Supplementary Table S7).

*Breast cancer-associated SNP in BMI ≥ 30 cohort.* Both BC-involved *MMP* polymorphic loci, c.836 A > G (rs17576) *MMP9*, and c. 1721 C > G (rs2250889) *MMP9*, are non-synonymous and lead to the replacement of amino acids (p.Q279R, and p.574P, respectively), in the MMP9 polypeptide with “benign/tolerated” PolyPhen-2/SIFT prediction classes.

In accordance with HaploReg publically available data, c.836 A > G (rs17576) *MMP9*, and c. 1721 C > G (rs2250889) *MMP9* are disposed in functionally active genome areas (DNase sensitive “open” chromatin) and affect allele-specific binding to transcription factors Pax-4 (rs17576) and NRSF (rs2250889) (Supplementary Table S5), regulatory protein CTCF (rs2250889), and transcription activity (due to the promoter/enhancer regulatory sequences). Importantly, c.836 A > G (rs17576) *MMP9* is situated in a DNA promoter site in adipose nuclei and mesenchymal stem cell-derived adipocyte-cultured cells, and enhancer regions in adipose nuclei. Along with this, c. 1721 C > G (rs2250889) *MMP9* is presented in enhancer regulatory sequence in adipose nuclei and adipose-derived mesenchymal stem cell-cultured cells.

The materials obtained from the GTE x portal database indicate the connection of both considered loci with transcription of the 13 genes *ZSWIM1*, *ZNF335*, *WFDC3*, *SPATA25*, *SLC12A5*, *RP11-465L10.10*, *RP3-337O18.9*, *MMP9*, *CD40*, *DNTTIP1* (rs17576), and *PCIF1*, *PLTP*, and *NEURL2* (rs17576 and rs2250889) (Supplementary Table S6). Among the above-mentioned 13 genes, 7 genes are expressed in adipose tissue (only rs17576 is adipose eQTL impact) such as adipose–subcutaneous (*NEURL2*, *PLTP*, *RP3-337O18.9*, *CD40*, *SLC12A5*) and adipose–visceral (omentum) (*NEURL2*, *ZSWIM1*, *PLTP*, *RP3-337O18.9*, *SPATA25*, *CD40*). It is important to emphasize that the BC risk allele c.836 A (rs17576) *MMP9* (according to our data, the reference allele G of this SNP is protective) determines the high transcriptional activity (β > 0) of the overwhelming number of the abovementioned genes (five out of seven); only the adipose expression of two genes (*SLC12A5*, and *CD40*) is negatively associated with this allele (β < 0). The splicing level of *SLC12A5* is linked with c.836 A > G (rs17576) *MMP9*, and c. 1721 C > G (rs2250889) *MMP9* (Supplementary Table S7).

## 4. Discussion

In the present study, we found obesity-specific associations of *MMP* gene polymorphic loci with BC: c.-1306 C > T (rs243865) *MMP2*, and c. 1331-163 G > A (rs3787268) *MMP9* were disease-linked among BMI < 30 subjects, and c.836 A > G (rs17576) *MMP9*, and c. 1721 C > G (rs2250889) *MMP9* were disorder-correlated among BMI ≥ 30 women. Based on in silico bioinformatics, we established pronounced functional effects of these loci (eQTL/sQTL/epigenetic) in adipose tissue.

Presently, the involvement of BMI in the predisposition to BC has been proven in numerous scientific studies [20,21,22,23,24], but at the same time there is an obvious inconsistency in the results obtained. On the one hand, the risk value of increased BMI for BC has been convincingly shown in large-scale epidemiological studies (including meta-analyses) [20,21,22,23]. On the other hand, there is no less convincing evidence of a negative association of BMI with BC [22,24]. A hypothesis is put forward about the risk role for BC of a higher BMI in postmenopausal women, whereas in premenopausal women, on the contrary, an increased BMI is a BC protective factor [23,24]. It is believed that postmenopausal women with a high BMI have larger fat reserves, which cause high concentrations of estrogens in the organism resulting in a BC risk factor [23,24,53]. However, a high BMI in premenopausal women determines a longer anovulatory cycle, which leads to low levels of progesterone and estrogen, resulting in a protective effect for BC in these women [23,24,54]. It should be noted that, in the sample of mostly postmenopausal women (2/3) we studied, a high BMI (BMI ≥ 30) is a risk factor for breast cancer (OR = 1.46, *p* = 0.01), which is completely consistent with the above literature data on this issue.

The results of previously performed associative studies on *MMP9* and *MMP2* polymorphic loci that showed a significant contribution to the BC susceptibility of obese/non-obese women are very ambiguous. The relation of c.-1306 C > T (rs243865) *MMP2* to BC was investigated in 17 experimental studies and three meta-analyses, among which only 8 studies (7 experimental studies and one meta-analysis) proved the risk (protective) value for the disease of the allelic variant C (T) (these results are completely consistent with our data in non-obese women), whereas in the vast majority of these studies, c.-1306 C > T (rs243865) was not associated with BC (Supplementary Table S1). The literature data for the c. 1331-163 G > A (rs3787268) locus of the *MMP9* gene are even more “confusing”: out of seven previously published papers on this topic (four experimental and three meta-analyses), four papers (two experimental [54,55] and two meta-analyses [56,57]) did not find its connection with the disease, two papers (experimental [16] and meta-analysis [8]) showed the protective effect of allele A, and Slattery et al. [58] and Fu et al. [59] demonstrated the risky effect of allele A on BC among predominantly (by 71–100%) Native American women and its association with poor disease-free survival of BC women, respectively, (the disease risk potential of the allele A in non-obese subjects is also shown in our work).

There is a similar “uncertainty” in the literature on the other two loci associated with BC in obese women, c.836 A > G (rs17576) *MMP9*, and c. 1721 C > G (rs2250889) *MMP9.* Relating to locus c. 1721 C > G (rs2250889) *MMP9*, there were previously seven publications (four experimental [17,55,59,60] and three meta-analyses [8,57,58]) and only in two of them (experimental [17] and meta-analysis [58]) associations of this polymorphism with the disease are shown (the G allele increased the BC risk; in our work, the opposite results were obtained; this allele has a protective effect for BCin obese women). The literature data on the association of the SNP c.836 A > G (rs17576) *MMP9* with BC are as follows: multidirectional data (protection/risk effects of SNP) are shown by Resler et al. [19] on the one hand (the BC protective effect of the G allele in obese women was also registered in our study) and by Chahil et al. [17] and Oliveira et al. [18]; on the other hand, in two experimental works [55,59] and in three meta-analyses [8,57,58] reliable connections of c.836 A > G (rs17576) *MMP9* with BC were not found.

Amongst the many possible reasons underlying the serious differences in the association character of the above *MMP* polymorphisms with BC in the foregoing works (ethnic and national factors, environmental factors, lifestyle, etc.), one of the causes may be the different premenopausal/postmenopausal statuses of studied subjects and, accordingly, the different BMI-BC links [20,23,24,53,54], as our research has shown various BMI-mediated *MMP*-BC associations. It should be noted that *MMP* genes (and the proteins of the same name controlled by them), by virtue of their strongly pronounced polyfunctional effects in the organism (due to the modification of the extracellular matrix), are pleiotropic genes and can affect not only BC but also the processes occurring in adipose tissue and BMI-related phenotypes (blood pressure, endothelial dysfunction, metabolic syndrome, etc.) [25,26,27,28,29], which ultimately also may define the *MMP*-triggered modifying influence of obesity on *MMP* associations with BC.

We found in silico substantial multidirectional influences on gene expression in adipose tissue BC-related polymorphic loci: BC risk allele c. 1331-163 A (rs3787268) in non-obese women is associated with low expression *NEURL2*, *PLTP*, *RP3-337O18.9*, *SPATA25*, and *ZSWIM1*, whereas BC risk allele c.836 A (rs17576) in obese women is associated with high expression of the same genes in visceral and/or subcutaneous adipose. Thus, according to our obtained data, women with a relatively low content of adipose tissue (BMI < 30) and low expression of the above-mentioned genes in adipose tissue will have an extremely minimal content of protein products of these genes, which significantly increases the BC risk in these women. On the contrary, women with excessive fat content (BMI ≥ 30) and high production of the same genes will have the maximum content of protein products of the above genes, which causes a significant BC risk increase in these women’s cohort.

Importantly, the aforementioned adipose-specifically-expressed genes are impactful for cancer biology. For example, the important clinical and pathobiological significance of the *ZSWIM1* gene (*SWIM-type zinc finger protein 1*) was shown in uterine endometrial carcinoma: the expression of this gene in the tumor was lower than in normal tissue, but the high expression of this gene negatively correlated with overall survival; the *ZSWIM1* methylation level was downregulated in cancer [61]. Pawar et al. showed upregulation of the *ZSWIM1* gene in ovarian cancer [62]. The previous study results demonstrated the substutional role of the *ZSWIM1* gene as a T helper cell (Th1) development/function regulator [63]. These immune system cells are responsible for tumor-significant immunity [62]. The *PLTP* gene-encoding phospholipid transfer protein promotes proliferation of the gastric cancer cell and this gene expression (mRNA and protein) may be a marker of gastric cancer progression/prognosis [64]. A high PLTP protein expression level is registered in clear cell renal cell carcinoma patients [65] and is a growth/migration stimulator of glioma cells [66]. According to the literature data, *NEURL2* (encodes neuralized U3-ubiquitin protein ligase 2) is a gene of the stem cells’ asymmetric division, and is involved in the self-renewal processes of mouse stemness and lung adenocarcinoma formation both in vitro and in vivo [67].The *NEURL2* gene is cancer-important; it may be a biomarker for clear cell renal cell carcinoma [68], a targeted gene for colorectal cancer [69], and as a result of the expression profile analysis, it was found that the region *NEURL2* gene may probably harbor a BC candidate gene [70].

The limitation of this study is the lack of experimental confirmation of the assumption put forward by us on the basis of in silico analysis about the multidirectional links of adipose eQTL effects of BC associated loci with the disease risk in obese and non-obese women.

## 5. Conclusions

The present study demonstrated the modifying effect of obesity on the association of *MMP* gene polymorphisms with BC: rs17576, and rs2250889 *MMP9* were BC protective factors in obese women; rs243865 *MMP2*, and rs3787268 *MMP9* determined BC susceptibility in non-obese women. We found in silico substantial multidirectional influences on gene expression in adipose tissue BC-related SNPs: BC risk allele A-rs3787268 in non-obese women associated with low expression *NEURL2*, *PLTP*, *RP3-337O18.9*, *SPATA25*, and *ZSWIM1*, whereas BC risk allele A-rs17576 in obese women associated with high expression of the same genes in visceral and/or subcutaneous adipose.

## Figures and Tables

**Table 1:** Phenotypic characteristics of the study participants.

Parameters	BMI ≥ 30			BMI < 30		
	BC Patients $\bar{X}$ ± SD/% (n)	Controls $\bar{X}$ ± SD/% (n)	*p*	BC Patients $\bar{X}$ ± SD/% (n)	Controls $\bar{X}$ ± SD/% (n)	*p*
*N*	119	190	-	239	556	-
Age, years (min–max)	58.97 ± 10.67 (33–84)	58.32 ± 10.08 (31–82)	0.47	53.58 ± 13.12 (28–82)	53.14 ± 12.68 (30–80)	0.63
<50 years	26.89 (32)	28.42 (54)	0.87	37.24 (89)	38.49 (214)	0.8
≥50 years	73.11 (87)	71.58 (136)		62.76 (150)	61.51 (342)	
BMI, kg/m^2^	34.95 ± 4.76	33.66 ± 4.12	0.007	27.55 ± 2.85	26.54 ± 2.71	0.01
Age at menarche, years	12.11 ± 1.02	12.32 ± 1.08	0.69	12.57 ± 1.05	12.73 ± 1.07	0.72
Age at menopause, years	48.58 ± 4.13	48.25 ± 4.02	0.63	48.08 ± 4.07	47.88 ± 4.01	0.74
Mensuration status						
premenopause	24.37 (29)	26.32 (50)	0.8	35.56 (85)	36.69 (204)	0.82
postmenopause	75.63 (90)	73.68 (140)		64.44 (154)	63.31 (352)	
Smoker (yes)	20.17 (24)	15.26 (29)	0.34	23.01 (55)	19.96 (111)	0.38
Biochemical parameters						
Fasting blood glucose (mmol/L)	8.76 ± 0.89	8.13 ± 0.79	<0.001	6.17 ± 0.75	5.23 ± 0.72	<0.001
TC (mmol/L)	6.34 ± 1.10	5.94 ± 1.05	<0.001	5.26 ± 1.01	4.85 ± 0.94	<0.001
HDL-C (mmol/L)	1.13 ± 0.45	1.25 ± 0.35	<0.001	1.40 ± 0.40	1.48 ± 0.41	<0.001
LDL-C (mmol/L)	4.31 ± 0.95	4.02 ± 0.88	<0.001	3.39 ± 0.79	3.11 ± 0.75	<0.001
TG (mmol/L)	1.98 ± 1.03	1.76 ± 1.01	<0.001	1.38 ± 0.64	1.23 ± 0.56	<0.001
Clinicopathological parameters of BC patients						
Stage of the cancer	T_0_-T_2_—79%, T_3_-T_4_—21%			T_0_-T_2_—72%, T_3_-T_4_—28%		
Lymph node involvement (N)	negative—50%, positive—50%			negative—46%, positive—54%		
Estrogen receptor (ER)	negative—31%, positive—69%			negative—36%, positive—64%		
Progesterone receptor (PR)	negative—38%, positive—62%			negative—43%, positive—57%		
Human epidermal growth factor receptor 2 (HER2)	negative—60%, positive—40%			negative—66%, positive—34%		
Tumor histological type	ductal—95%, lobular—5%			ductal—94%, lobular—6%		
Tumor histological grade (G)	G1/G2—70%, G3—30%			G1/G2—67%, G3—33%		
Progression	absent—68%, present—32%			absent—65%, present—35%		
Metastasis	absent—80%, present—20%			absent—77%, present—23%		
Death	absent—76%, present—24%			absent—83%, present—17%		

Note: TC—total cholesterol; HDL-C—high-density lipoprotein cholesterol; LDL-C—low-density lipoprotein cholesterol; TG—triglyceride; G1–well-differentiated; G2–moderately differentiated; G3–poorly differentiated.

**Table 2:** Associations of the studied gene polymorphisms with breast cancer among BMI < 30 and BMI ≥ 30 females.

Chr	SNP	Gene	Minor Allele	n	Allelic Model				Additive Model				Dominant Model				Recessive Model			
					OR	95% CI		*p*	OR	95% CI		*p*	OR	95% CI		*p*	OR	95% CI		*p*
						L95	U95			L95	U95			L95	U95			L95	U95	
**Female with BMI < 30**																				
11	rs1940475	*MMP-8*	T	778	0.94	0.75	1.16	0.546	1.04	0.79	1.37	0.795	0.92	0.59	1.45	0.729	1.20	0.76	1.89	0.432
11	rs1799750	*MMP-1*	2G	763	1.00	0.80	1.24	0.999	1.09	0.81	1.46	0.562	1.22	0.77	1.94	0.390	1.01	0.61	1.67	0.972
11	rs679620	*MMP-3*	T	778	0.86	0.69	1.07	0.165	0.80	0.59	1.07	0.126	0.78	0.50	1.23	0.288	0.69	0.42	1.14	0.151
16	rs243865	*MMP-2*	T	767	0.95	0.74	1.22	0.675	0.79	0.56	1.12	0.191	0.87	0.57	1.32	0.518	**0.31**	**0.09**	**0.99**	**0.043**
20	rs3918242	*MMP-9*	T	775	0.95	0.70	1.28	0.730	0.97	0.64	1.45	0.863	0.95	0.60	1.05	0.823	1.06	0.28	3.95	0.936
20	rs3918249	*MMP-9*	C	771	0.90	0.72	1.13	0.350	1.02	0.76	1.36	0.922	0.97	0.64	1.47	0.886	1.12	0.64	1.96	0.698
20	rs17576	*MMP-9*	G	778	0.87	0.69	1.09	0.222	0.85	0.63	1.15	0.303	0.88	0.58	1.34	0.559	0.67	0.36	1.28	0.225
20	rs3787268	*MMP-9*	A	770	1.25	0.97	1.61	0.088	1.36	0.98	1.88	0.065	1.28	0.85	1.93	0.240	**2.36**	**1.12**	**4.97**	**0.024**
20	rs2250889	*MMP-9*	G	772	0.79	0.54	1.14	0.207	0.72	0.43	1.19	0.198	0.65	0.37	1.15	0.138	1.07	0.23	5.03	0.936
20	rs17577	*MMP-9*	A	766	0.98	0.73	1.32	0.912	1.05	0.70	1.56	0.829	1.05	0.67	1.65	0.838	1.09	0.28	4.16	0.906
**Female with BMI ≥ 30**																				
11	rs1940475	*MMP-8*	T	307	1.07	0.77	1.48	0.693	1.06	0.77	1.45	0.736	1.06	0.63	1.76	0.839	1.10	0.64	1.89	0.720
11	rs1799750	*MMP-1*	2G	303	1.05	0.75	1.45	0.785	1.06	0.77	1.46	0.701	1.29	0.76	2.16	0.345	0.91	0.53	1.57	0.734
11	rs679620	*MMP-3*	T	306	1.00	0.72	1.39	1.000	1.05	0.75	1.47	0.774	1.01	0.64	1.90	0.732	1.04	0.60	1.78	0.900
16	rs243865	*MMP-2*	T	304	0.92	0.62	1.37	0.695	0.98	0.66	1.45	0.903	1.03	0.64	1.67	0.894	0.72	0.24	2.11	0.547
20	rs3918242	*MMP-9*	T	303	1.09	0.72	1.63	0.694	1.06	0.70	1.60	0.797	1.00	0.61	1.63	0.996	1.57	0.48	5.08	0.453
20	rs3918249	*MMP-9*	C	301	0.83	0.59	1.16	0.271	0.83	0.60	1.16	0.282	0.77	0.47	1.24	0.281	0.81	0.42	1.54	0.522
20	rs17576	*MMP-9*	G	306	**0.71**	**0.50**	**1.00**	**0.047**	0.72	0.52	1.01	0.060	0.67	0.42	1.07	0.095	0.61	0.31	1.20	0.156
20	rs3787268	*MMP-9*	A	308	0.86	0.57	1.30	0.469	0.86	0.56	1.32	0.486	0.80	0.49	1.29	0.358	1.29	0.33	4.98	0.711
20	rs2250889	*MMP-9*	G	307	0.56	0.31	1.02	0.054	**0.55**	**0.31**	**0.98**	**0.042**	0.53	0.27	1.01	0.055	0.26	0.03	2.26	0.221
20	rs17577	*MMP-9*	A	317	0.89	0.58	1.34	0.569	0.86	0.57	1.30	0.468	0.84	0.51	1.38	0.494	0.78	0.25	2.42	0.662

Note: OR–odds ratio; 95% CI—95% confidence interval; All results were obtained after adjustment for covariates; *p* values < 0.05 are shown in bold.

## Data Availability

The data generated in the present study are available from the corresponding author upon reasonable request.

## Supplementary Materials

The following supporting information can be downloaded at: https://www.mdpi.com/article/10.3390/biomedicines10102617/s1, Table S1: The literature data about associations of the studied polymorphisms of the MMP genes with breast cancer; Table S2: The regulatory potential of the studied SNPs; Table S3: The allele and genotype frequencies of the studied SNPs in the breast cancer and control groups with BMI < 30; Table S4: The allele and genotype frequencies of the studied SNPs in the breast cancer and control groups with BMI ≥ 30; Table S5: Effect of the BC-associated *MMP* gene polymorphisms on affinity of the DNA regulatory motifs; Table S6: eQTL values of the BC-associated SNPs of the *MMP* genes; Table S7: sQTL values of the BC-associated SNPs of the *MMP* genes.
