# Supplementary material for: The Modifying Effect of Obesity on the Association of Matrix Metalloproteinase Gene Polymorphisms with Breast Cancer Risk
Source: Biomedicines. 2022 Oct 18;10(10):2617. doi: 10.3390/biomedicines10102617 (PMC9599943; doi:10.3390/biomedicines10102617)
Supplement: Supplementary file 1 [file biomedicines-10-02617-s001.zip › Supplementary Table S3.pdf]

Supplementary Table S3

The allele and genotype frequencies of the studied SNPs in the breast cancer and control groups with BMI<30.

Chr	SNP	Gene	Minor allele	Major allele	Minor allele frequency	Number of the studied chromosomes	Genotype distribution *	H_o	H_e	P_{HWE}
Breast cancer patients (n=239)										
11	rs1940475	*MMP-8*	T	C	0.479	474	46/135/56	0.57	0.50	0.037
11	rs1799750	*MMP-1*	2G	1G	0.460	474	48/122/67	0.51	0.50	0.604
11	rs679620	*MMP-3*	T	C	0.460	476	48/123/67	0.52	0.50	0.602
16	rs243865	*MMP-2*	T	C	0.247	466	12/91/130	0.39	0.37	0.596
20	rs3918242	*MMP-9*	T	C	0.150	474	9/53/175	0.22	0.25	0.071
20	rs3918249	*MMP-9*	C	T	0.358	466	34/99/100	0.42	0.46	0.255
20	rs17576	*MMP-9*	G	A	0.351	470	33/99/103	0.42	0.46	0.253
20	rs3787268	*MMP-9*	A	G	0.257	464	19/81/132	0.35	0.38	0.227
20	rs2250889	*MMP-9*	G	C	0.087	472	3/35/198	0.15	0.16	0.395
20	rs17577	*MMP-9*	A	G	0.156	474	9/56/172	0.24	0.26	0.134
Control group (n=556)										
11	rs1940475	*MMP-8*	T	C	0.496	1104	149/249/154	0.45	0.50	0.022
11	rs1799750	*MMP-1*	2G	1G	0.460	1074	119/256/162	0.48	0.50	0.341
11	rs679620	*MMP-3*	T	C	0.498	1102	141/267/143	0.48	0.50	0.495
16	rs243865	*MMP-2*	T	C	0.257	1090	40/200/305	0.37	0.38	0.370
20	rs3918242	*MMP-9*	T	C	0.157	1098	12/148/389	0.27	0.26	0.747
20	rs3918249	*MMP-9*	C	T	0.383	1098	80/261/208	0.48	0.47	0.928
20	rs17576	*MMP-9*	G	A	0.384	1108	84/257/213	0.46	0.47	0.654
20	rs3787268	*MMP-9*	A	G	0.217	1098	25/188/336	0.34	0.34	0.901
20	rs2250889	*MMP-9*	G	C	0.108	1094	10/98/439	0.18	0.19	0.117
20	rs17577	*MMP-9*	A	G	0.158	1080	11/149/380	0.28	0.27	0.518

Note: * minor allele homozygotes / heterozygotes / major allele homozygotes.
