# Supplementary material for: The Modifying Effect of Obesity on the Association of Matrix Metalloproteinase Gene Polymorphisms with Breast Cancer Risk
Source: Biomedicines. 2022 Oct 18;10(10):2617. doi: 10.3390/biomedicines10102617 (PMC9599943; doi:10.3390/biomedicines10102617)
Supplement: Supplementary file 1 [file biomedicines-10-02617-s001.zip › Supplementary Table S4.pdf]

Supplementary Table S4

The allele and genotype frequencies of the studied SNPs in the breast cancer and control groups with BMI≥30.

Chr	SNP	Gene	Minor allele	Major allele	Minor allele frequency	Number of the studied chromosomes	Genotype distribution *	H_o	H_e	P_{HWE}
Breast cancer patients (n=119)										
11	rs1940475	*MMP-8*	T	C	0.487	236	30/55/33	0.47	0.50	0.465
11	rs1799750	*MMP-1*	2G	1G	0.487	234	28/58/31	0.50	0.50	1.000
11	rs679620	*MMP-3*	T	C	0.500	236	29/60/29	0.51	0.50	1.000
16	rs243865	*MMP-2*	T	C	0.214	234	5/40/72	0.34	0.34	1.000
20	rs3918242	*MMP-9*	T	C	0.209	234	6/37/74	0.32	0.33	0.583
20	rs3918249	*MMP-9*	C	T	0.361	230	17/49/49	0.43	0.46	0.422
20	rs17576	*MMP-9*	G	A	0.325	234	14/48/55	0.41	0.44	0.527
20	rs3787268	*MMP-9*	A	G	0.186	236	4/36/78	0.31	0.30	1.000
20	rs2250889	*MMP-9*	G	C	0.067	238	1/14/104	0.12	0.13	0.417
20	rs17577	*MMP-9*	A	G	0.185	232	5/33/78	0.28	0.30	0.539
Control group (n=190)										
11	rs1940475	*MMP-8*	T	C	0.471	378	45/88/56	0.47	0.50	0.382
11	rs1799750	*MMP-1*	2G	1G	0.476	372	49/79/58	0.42	0.50	0.041
11	rs679620	*MMP-3*	T	C	0.500	376	47/94/47	0.50	0.50	1.000
16	rs243865	*MMP-2*	T	C	0.227	374	12/61/114	0.33	0.35	0.305
20	rs3918242	*MMP-9*	T	C	0.196	372	6/61/119	0.33	0.32	0.816
20	rs3918249	*MMP-9*	C	T	0.406	372	33/85/68	0.46	0.48	0.543
20	rs17576	*MMP-9*	G	A	0.405	378	35/83/71	0.44	0.48	0.229
20	rs3787268	*MMP-9*	A	G	0.211	380	5/70/115	0.37	0.33	0.189
20	rs2250889	*MMP-9*	G	C	0.114	376	5/33/150	0.18	0.20	0.072
20	rs17577	*MMP-9*	A	G	0.204	372	9/58/119	0.31	0.33	0.651

Note: * minor allele homozygotes / heterozygotes / major allele homozygotes.
